# Lnc‐ANRIL modulates the immune response associated with NF‐κB pathway in LPS‐stimulated bovine mammary epithelial cells

**DOI:** 10.1002/iid3.1125

**Published:** 2023-12-22

**Authors:** Jinye Lu, Beibei Gu, Wei Lu, Jing Liu, Jiang Lu

**Affiliations:** ^1^ College of Pet Technology Jiangsu Agri‐Animal Husbandry Vocational College Taizhou China; ^2^ Integrated Technical Service Center Taizhou Customs Taizhou China

**Keywords:** immune response, Lnc‐ANRIL, NF‐κB pathway

## Abstract

**Background:**

The antisense noncoding RNA in the INK4 locus (ANRIL) has been confirmed related to multiple disease progression, but the role and exact mechanisms of lnc‐ANRIL in lipopolysaccharide (LPS)‐induced inflammation of bovine mammary epithelial cells (MAC‐T) remain unclear.

**Aims:**

This manuscript focused on expounding the functional role of lnc‐ANRIL through experiments performed in MAC‐T.

**Methods:**

At the in vitro level, we established a Bovine mammary epithelial cell (BMEC) cell model of mastitis by LPS treatment. Transfection of siRNA was examined by immunofluorescence localization and RT‐qPCR. CCK8, clonogenic assay and EdU were used to detect the proliferation ability of the cells. Cell cycle and apoptosis were detected by flow cytometry and Western blot. The levels of inflammatory factors and oxidative stress markers were detected by ELISA kits.

**Results:**

Cell Counting Kit‐8, colony formation, and 5‐ethynyl‐20‐deoxyuridine were adopted and the data illustrated that LPS could significantly suppress the cell proliferation, while knockdown of lnc‐ANRIL expression obviously promoted MAC‐T cell proliferation compared with LPS or LPS + si‐NC group. Flow cytometry analysis demonstrated that lnc‐ANRIL could induce MAC‐T cell apoptosis. In addition, downregulation of lnc‐ANRIL affected LPS‐induced immune response by regulating inflammatory factor expressions and modulating the nuclear factor kappa B (NF‐κB) axis in MAC‐T cells.

**Conclusion:**

Our results suggest that lnc‐ANRIL is involved in the regulation of cell proliferation, cell cycle, and cell apoptosis of MAC‐T cells, and plays an important role in the inflammatory and immune response of MAC‐T cells through the regulation of the NF‐κB pathway, proposing new therapeutic strategies for the treatment of innate immune response‐related disease such as bovine mastitis.

## Graphical abstract

Silencing of Inc‐antisense noncoding RNA in the INK4 locus (ANRIL) expression could inhibit inflammatory response by regulating nuclear factor kappa B (NF‐κB) signaling pathway. Lnc‐ANRlL exerted vital erects during very first stages of innate immune response in mammary epithelial cells. Our findings proposed new therapeutic strategies for the treatment or innate immune response‐related diseases such as bovine mastitis.

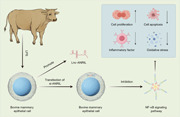

## 1 INTRODUCTION

Bovine mammary epithelial cell (BMEC) exhibits the breast cell types and responses to gram‐negative bacterial lipopolysaccharide (LPS) stimulation to further initiate the pathogen‐associated molecular changes through activating various pattern recognition receptors, leading to cascades of transcriptional regulatory events in a different order, resulting in inflammation and oxidative stress.^1^, ^2^, ^3^ Bovine mastitis is an important factor affecting milk quality and food safety.^4^ Drug residues and drug resistance are easy to occur in the current drug treatment methods for dairy bovine mastitis^5^ Effective research of the pathogenesis can provide new insights into the development of treatment strategies for bovine mastitis.

Long noncoding RNAs (lncRNAs), as a kind of noncoding RNA with more than 200 nT lengths, are located in the nucleus and cytoplasm,^6^, ^7^ and exert critical effects on the physiological and pathological processes through regulating the expression of related genes.^8^, ^9^ Of note, accumulating evidence suggests that lncRNAs are involved in inflammatory progression in the BMECs (MAC‐T). For example, lncRNA H19 and lncRNA‐TUB upregulation influence BMEC characteristics and immune responses.^10^, ^11^ LncRNA XIST modulates NF‐κB/NLRP3 signaling to affect BMEC inflammatory responses.^12^

Antisense noncoding RNA in the INK4 locus (ANRIL), located on chromosome 9p21, is identified in familial melanoma patients. Recently, lnc‐ANRIL participated in the prognosis of sepsis, indicating the essential role in systemic inflammation. For instance, lncRNA ANRIL affects osteoarthritis synoviocyte proliferation and apoptosis in osteoarthritis progression.^13^ LncRNA ANRIL acts as a biomarker for bronchial asthma.^14^ Moreover, lncRNA ANRIL activates NLRP3 inflammasome in uric acid nephropathy.^15^ In an Alzheimer's disease PC12 cell model, lncRNA ANRIL knockdown inhibits apoptosis and inflammatory responses while promoting neurite growth by binding to miR‐125a.^16^ LncRNA ANRIL has been shown to play a role in the pathogenesis of immune‐related diseases, such as inflammatory bowel disease, coronary artery disease, type 2 diabetes, and cancer.^17^, ^18^ However, detailed impacts of lnc‐ANRIL and possible mechanisms on the pathogenesis of bovine mastitis remain unknown. This study aims to elucidate the potential impacts of lnc‐ANRIL on MAC‐T and explore potential mechanisms, thereby offering a novel therapeutic approach for bovine mastitis treatment.

## 2 MATERIALS AND METHODS

### 2.1 Ethics approval and consent to participate

The authors affirm their adherence to the ethical policies of the journal, as outlined on the author guidelines page of the journal. No ethical approval was required as this study excluded experiments about people or animal tissues.

### 2.2 Cell culture and transfection

Bovine MAC‐T were provided by ATCC (USA) and came with comprehensive authentication and quality controls. MAC‐T cells were resuscitated and passaged three times before experiments. The cells were then cultured within DMEM/F12 (Thermo Fisher Scientific) that contained 10% fetal bovine serum (Gibico) and incubated within the humid incubator under 37°C and 5% CO_2_ conditions, along with the change of medium every 3 days. Cultured MAC‐T cells were seeded in 6‐well plates and treated with 100 ng/mL LPS (Gibco)^19^ for 24 h to induce an inflammatory cell model after 70%–80% confluence.

Genechem was responsible for constructing si‐ANRIL and corresponding negative control (si‐NC). All RNA vectors were labeled with a green fluorescent protein (GFP). According to the instruction, 2 µg si‐ANRIL (5′‐TGGATCCCAACAGACTCAACCGCTT‐3′) or si‐NC (5′‐TTCTCCGAACGTGTCACGT‐3′) was individually transfected into MAC‐T cells via Lipofectamine™ 3000 (Thermo Fisher Scientific). The transfection efficiency was measured by GFP expression and reverse transcription‐quantitative polymerase chain reaction (RT‐qPCR) after transfection for 24 h at 37°C.

### 2.3 Cell Counting Kit‐8 (CCK‐8) assay

Treated MAC‐T cells (1 × 10^4^/well) from different groups were inoculated into 96‐well plates. After incubation for 0, 24, 48, and 72 h, 10 µL of CCK‐8 solution (Sigma) was added to each well and incubated at 37°C, 5% CO_2_ for 3 h. Absorbance (OD = 450 nm) was detected using a spectrophotometer (Molecular Devices).

### 2.4 Cell proliferation assay

The treated MAC‐T cells (1 × 10^4^/well) in different groups were seeded into 24‐well plates and cultured for 48 h in a 37°C 5% CO_2_ incubator. After being fixed with 4% paraformaldehyde, goat serum was used to block cells for another 1 h. Furthermore, the cells were stained with 0.1% crystal violet (Nakaraitesk) for 1 h at room temperature. Colonies were observed and counted under a light microscope.

5‐Ethynyl‐20‐deoxyuridine (EdU) assays were performed using an EdU staining kit (RiboBio). Treated MAC‐T cells (1 × 10^3^/well) from different groups were cultured in 6‐well plates, and the medium was replaced for a total of 2 weeks. Cells were then incubated with 50 µM EdU solution for 1 h at room temperature. Subsequently, cells were fixed with 4% neutral paraformaldehyde for 30 min at room temperature. Cells were permeabilized with PBS containing 0.5% Triton X‐100 for 20 min and then washed three times with PBS. Cells were stained with Apollo staining reagent (KeyGEN) for 20 min. Nuclei were stained with 4′, 6‐Diamidino‐2‐Phenylindole (KeyGen Institute of Biotechnology). Images were captured using a fluorescence microscope (CompixInc).

### 2.5 Flow cytometry analysis

Flow cytometry analysis was used to detect cell apoptosis. Treated MAC‐T cells from different groups were collected and rinsed. Then 5 μL of Annexin V‐FITC together with 10 μL of PI (Annexin V‐FITC Apoptosis Detection Kit, Immunostep) was utilized to treat MAC‐T cells for 15 min. A flow cytometer (BD Biosciences) was utilized for determining apoptotic cells.

The cell cycle was analyzed by flow cytometry. Treated MAC‐T cells from different groups were fixed with precooled 70% ethanol at 4°C overnight. After washing, 100 µL RNaseA (19101, Qiagen) mixture solution was added and incubated for 30 min at room temperature. Following that, a 400 μL PI dye solution (K201, BioVision Inc.) was added and placed in the greenhouse for 30 min. The cell cycle distribution was detected by a flow cytometer (BD Biosciences).

The gating strategy in flow cytometry is as follows. First, we need to select the target cell group based on forward scatter and side scatter parameters, which can exclude cell fragments and dead cells. Afterward, after adjusting the fluorescence compensation, the gate is set based on the signals of unstained control cells and individual positive staining cells.

### 2.6 Enzyme‐linked immunosorbent assay (ELISA)

To determine inflammatory cytokine levels, ELISA kits human interleukin‐1β (IL‐1β) (TWp023753, Shanghai Tongwei Industrial Co., Ltd.), IL‐6 (TWp023756, Shanghai Tongwei Industrial Co., Ltd.), tumor necrosis factor‐α (TNF‐α) (TWp024586, Shanghai Tongwei Industrial Co., Ltd.) were used. To determine the oxidative stress level, superoxide dismutase (SOD) (TW14946, Shanghai Tongwei Industrial Co., Ltd.), malondialdehyde (MDA) (HZ4709‐1, Shanghai Huzhen Biotechnology Co., Ltd.), and myeloperoxidase (MPO) (HZA601Bo, Shanghai Huzhen Biotechnology Co., Ltd.) were analyzed through ELISA in line with specific protocols.

### 2.7 Reverse transcription‐quantitative polymerase chain reaction (RT‐qPCR) analysis

TRIzol reagents (Beyotime Biotechnology) were utilized to extract total RNA from MAC‐T cells, and TaqMan one‐step reverse transcription (Applied Biosystems) was conducted for preparing cDNA. The ABI Prism 7500 system (Applied Biosystems) was used for RT‐qPCR following specific protocols. GAPDH served as the endogenous reference. The primer sequences were as follows: GAPDH forward 5′‐ACGGCACAGTCAAGGCAGA‐3′ and reverse 5′‐GTGATGGCGTGGACAGTGG‐3′; Lnc‐ANRIL forward 5′‐AGTTCGCCACCCCAACTTAG‐3′ and reverse 5′‐AAAGAAAGCGTTTGGTCGCC‐3′; p65 forward 5′‐CATCAGCCAGCGCATCCAGA‐3′ and reverse 5′‐TGGGGTGAGAGAGGACAGGC‐3′; IκBα forward 5′‐CCGGAATTCGAGCGGCCCCCGGGGCTG‐3′ and reverse 5′‐AAACTCGAGTTATTCTGTTAACCAACTCCAATC‐3′.

### 2.8 Western blot analysis assay

Protein was extracted from MAC‐T cells and measured through the bicinchoninic acid kit (Beyotime Biotechnology). Subsequently, the protein was extracted through the utilization of Sodium Dodecyl Sulfate Polyacrylamide Gel Electrophoresis (10%) and subsequently transferred onto polyvinyldifluoride membranes. Afterward, membranes were incubated using 5% skimmed milk, and incubated with primary antibodies under 4°C overnight. The antibodies are as follows: anti‐p65 (bs‐20160R, Bioss), anti‐p‐p65 (bs‐0982R, Bioss), anti‐IκBα (bs‐1287R, Bioss), anti‐p‐IκBα (bs‐18128R, Bioss), P21 (sc‐65595, Santa Cruz), P27 (sc‐71813, Santa Cruz), Cyclin D1 (sc‐246, Santa Cruz), caspase‐3 (bsm33199m, Bioss), caspase‐9 (bs‐20773R, Bioss), Bax (bs‐0127R, Bioss), Bcl‐2 (bsm‐33413M, Bioss), and anti‐GAPDH (bs‐0755R, Bioss), with GAPDH being the endogenous control. After three cleanings with 0.1% tween 20 (TBST) buffer, membranes were further incubated for 1 h using HRP‐labeled secondary antibody (1:4,000, SA00004‐10, Proteictech). Finally, the enhanced chemiluminescence kit (ECL) was utilized to observe protein blots, whereas ImageJ software (NIH, version 4.3) was adopted for quantification.

### 2.9 Immunohistochemical analysis

The cell slices from different groups were sealed for 30 min with 3% bovine serum protein at 37°C. Then slides were incubated overnight with primary antibodies anti‐p65 (bs‐20160R, Bioss), anti‐p‐p65 (bs‐0982R, Bioss), anti‐IκBα (bs‐1287R, Bioss), anti‐p‐IκBα (bs‐18128R, Bioss). The slices were subsequently subjected to incubation with a second antibody, Goat Anti‐Rabbit IgG H&L (HRP) (bs‐0295G‐HRP, Bioss), for 60 min at ambient temperature. Following this, the slices were washed three times with PBS, with each wash lasting 5 min. The PBS solution was removed, and 50 μL Diaminobenzidine solution was added to each section and rinsed with distilled water. Hematoxylin staining was performed for 25 s and blue was returned by rinsing with running water for 3 min. Fields were photographed in each slice using a microscope (Leica Microsystems).

### 2.10 Statistical analysis

Data are shown as the average ± standard deviation. All experiments were repeated at least three times independently. GraphPad Prism 5.0 software (GraphPad Software Inc.) was used for data analysis. *T* test was used for comparison between two groups, one‐way analysis of variance and Tukey's posttest were used for comparison between three or more groups, and a *p* < .05 was considered statistically significant.

## 3 RESULT

### 3.1 Knockdown of lnc‐ANRIL promotes cell proliferation of MAC‐T cells

**Figure 1:**
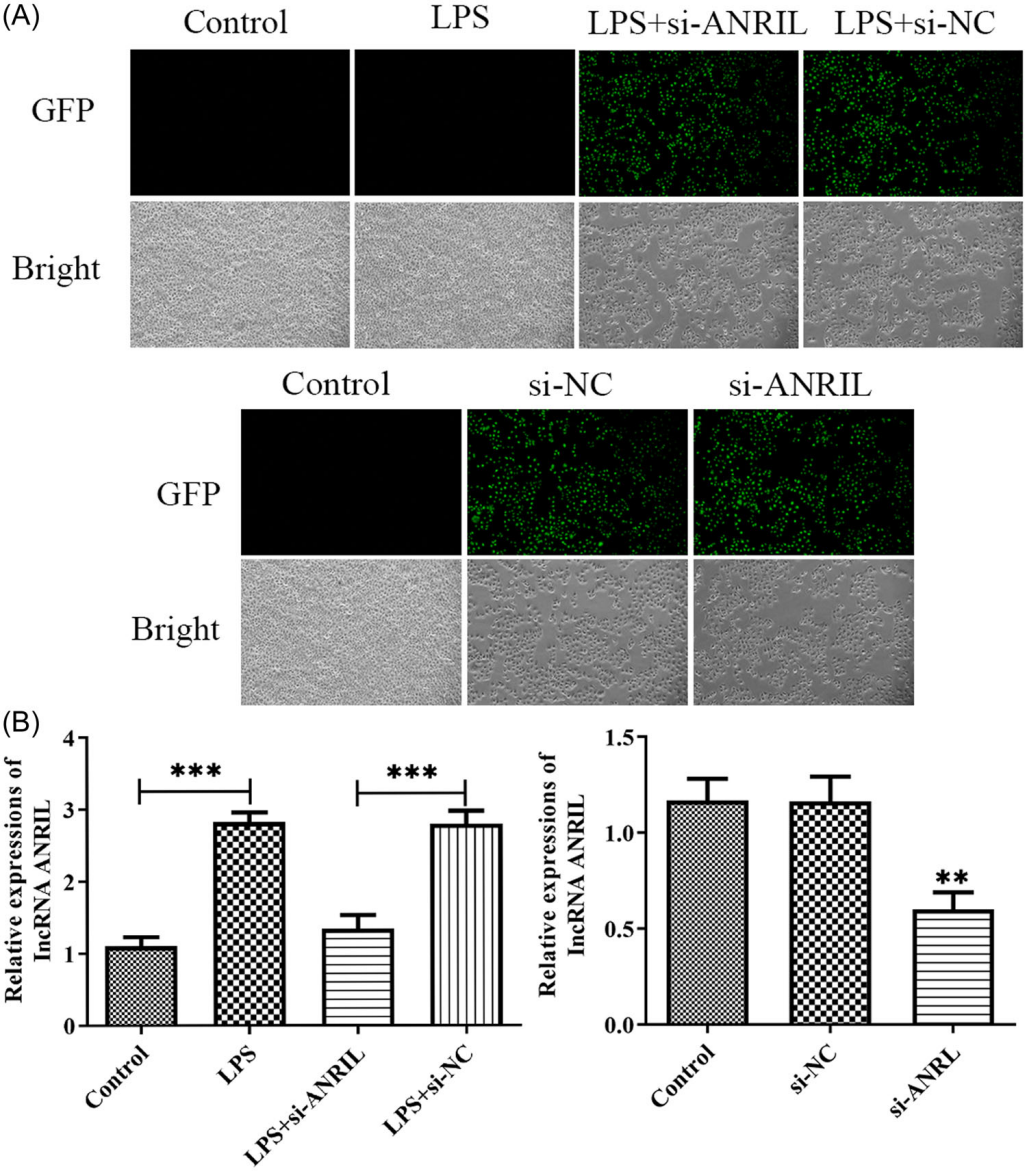
Analysis of transfection efficiency. (A) Immunofluorescence localization of green fluorescent protein confirmed successful transfection of siRNA. (B) RT‐qPCR assays were used to detect transfection efficiency. The experiments were replicated thrice and subjected to analysis using GraphPad Prism 5.0 software, employing analysis of variance in conjunction with Tukey's posthoc test. **p < .01, ***p < .001 compare with Control or lipopolysaccharide + si‐NC or si‐NC.

We want to explore the potential effects of lnc‐ANRIL on MAC‐T cells. MAC‐T cells induced with endotoxin‐LPS and transfected with siRNA‐ANRIL, and GFP and RT‐qPCR were conducted to assess transfection efficiency. In Figure 1A, the results of fluorescence microscopy showed that si‐ANRIL was successfully transfected into MAC‐T cells or LPS‐treated MAC‐T cells. Meanwhile, RT‐qPCR results showed that the expression level of lnc‐ANRIL in MAC‐T cells treated with LPS was significantly higher (*p* < .01) than that in the Control group. Compared with the LPS + si‐NC group, the expression level of lnc‐ANRIL in MAC‐T cells of the LPS + si‐ANRIL group was significantly decreased (*p* < .01). In addition, the expression level of lnc‐ANRIL in MAC‐T cells transfected with si‐ANRIL was significantly lower (*p* < .01) than that in the si‐NC group (Figure 1B).

**Figure 2:**
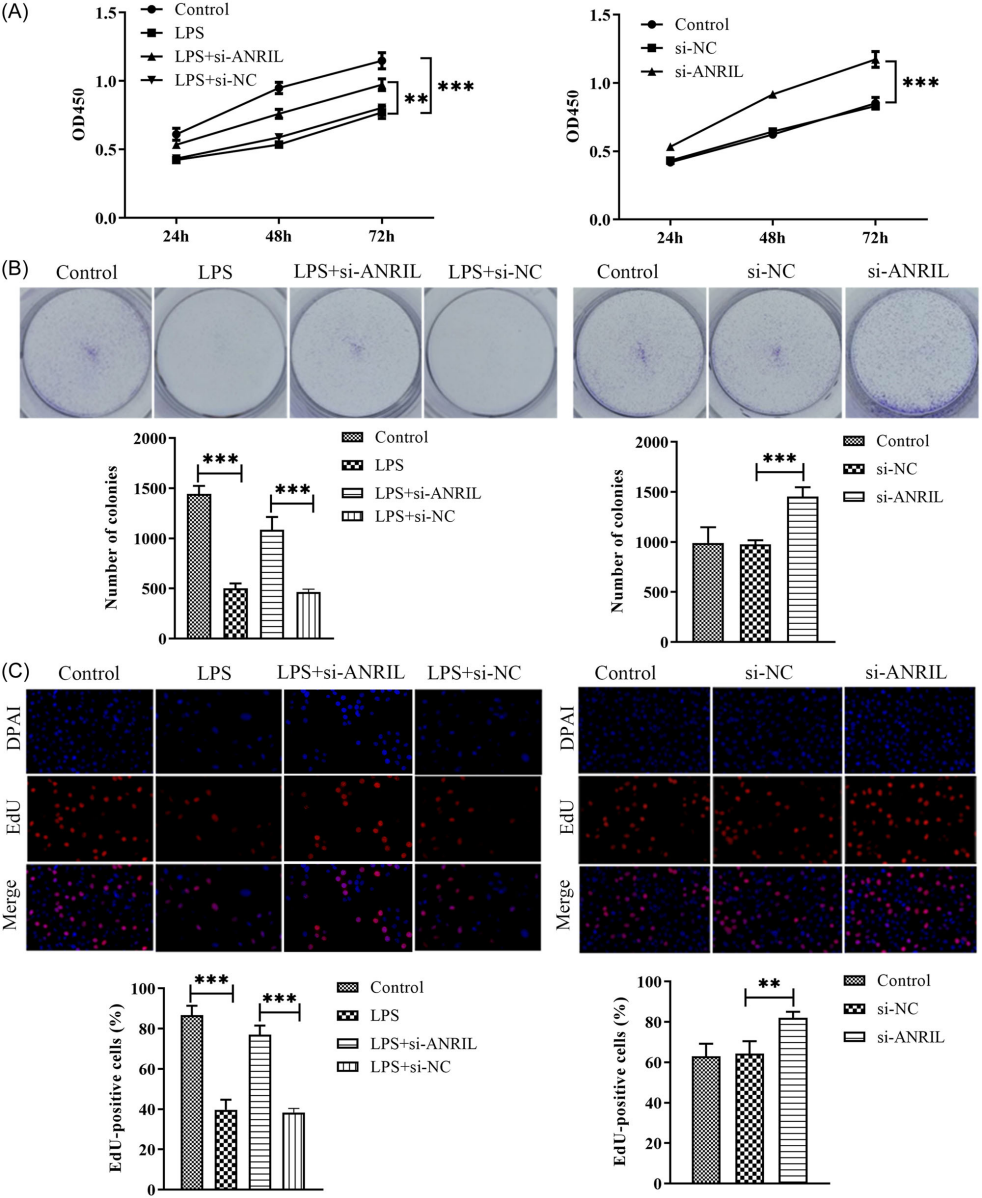
Knockdown of lnc‐antisense noncoding RNA in the INK4 locus (ANRIL) expression promoted cell proliferation of lipopolysaccharide (LPS)‐stimulated bovine mammary epithelial cells (MAC‐T). (A) Cell Counting Kit‐8 assay was performed to investigate the role of lnc‐ANRIL in the viability of MAC‐T. (B) A colony formation assay was used to determine the effects of lnc‐ANRIL on the colony formation abilities of MAC‐T cells. (C) 5‐Ethynyl‐20‐deoxyuridine assay was performed to assess the effects of lnc‐ANRIL on the proliferation of MAC‐T cells. The experiments were replicated thrice and subjected to analysis using GraphPad Prism 5.0 software, employing analysis of variance in conjunction with Tukey's posthoc test. **p < .01, ***p < .001 compare with Control or LPS + si‐NC or si‐NC.

Functionally, the CCK‐8 data showed that LPS could significantly inhibit the viability of MAC‐T cells compared with the control group (*p* < .01). In LPS‐induced cells, the viability of cells transfected with si‐ANRIL was significantly increased compared with those transfected with si‐NC (*p* < .01) (Figure 2A). Based on colony formation, LPS could significantly suppress the colony formation ability of MAC‐T cells compared with the control group. However, the knockdown of lnc‐ANRIL expression promoted the colony formation ability of MAC‐T cells compared with LPS and LPS + si‐NC group (Figure 2B). The result of EdU (Figure 2C) illustrated that the percentage of proliferative cells within the LPS + si‐ANRIL group was enhanced, indicating that lnc‐ANRIL downregulation promoted the proliferation of MAC‐T cells (Figure 2C). These findings suggested a negative association between lnc‐ANRIL levels and cell proliferation.

### 3.2 Silencing of lnc‐ANRIL expression reduces cell arrest

**Figure 3:**
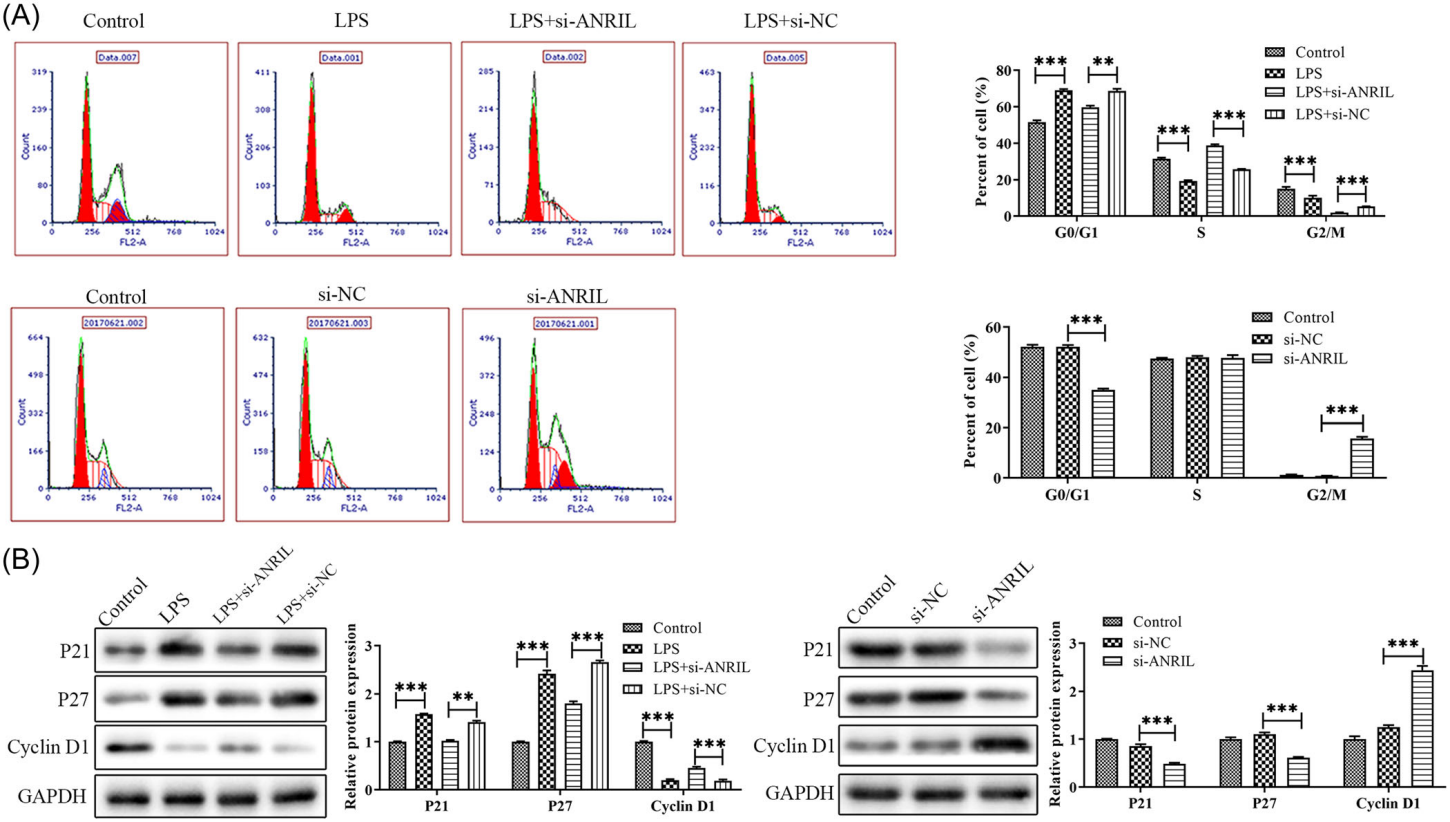
The effect of lnc‐antisense noncoding RNA in the INK4 locus (ANRIL) on cell cycle. (A) Flow cytometry was used to detect the activity of lnc‐ANRIL on the cell cycle. (B) The expression level of cell cycle‐related proteins P21, P27, and Cyclin D1 was assessed by western blot. The experiments were replicated thrice and subjected to analysis using GraphPad Prism 5.0 software, employing analysis of variance in conjunction with Tukey's posthoc test. **p < .01, ***p < .001 compare with Control or lipopolysaccharide + si‐NC or si‐NC.

Flow cytometry was carried out to detect lnc‐ANRIL effects on the cell cycle. As shown in Figure 3A, after LPS treatment, the cell cycle was arrested in the G0/G1 phase compared to the control group. Compared with the LPS+si‐NC group, LPS + si‐ANRIL treatment reduced cell cycle arrest in the G0/G1 phase. Compared with the si‐NC group, the proportion of cells in the G0/G1 phase in the si‐ANRIL group was significantly reduced. In Figure 3B, Western blot results showed that LPS significantly increased the expression levels of P21 and P27 proteins and decreased the expression levels of cyclin D1 proteins compared with the control group. LPS + si‐ANRIL group reversed this effect. Compared with the si‐NC group, the protein levels of P21 and P27 in the si‐ANRIL group were significantly decreased, and the expression level of cyclin D1 protein was increased in the si‐ANRIL group.

### 3.3 Knockdown of lnc‐ANRIL inhibits cell apoptosis of bovine MAC‐T

**Figure 4:**
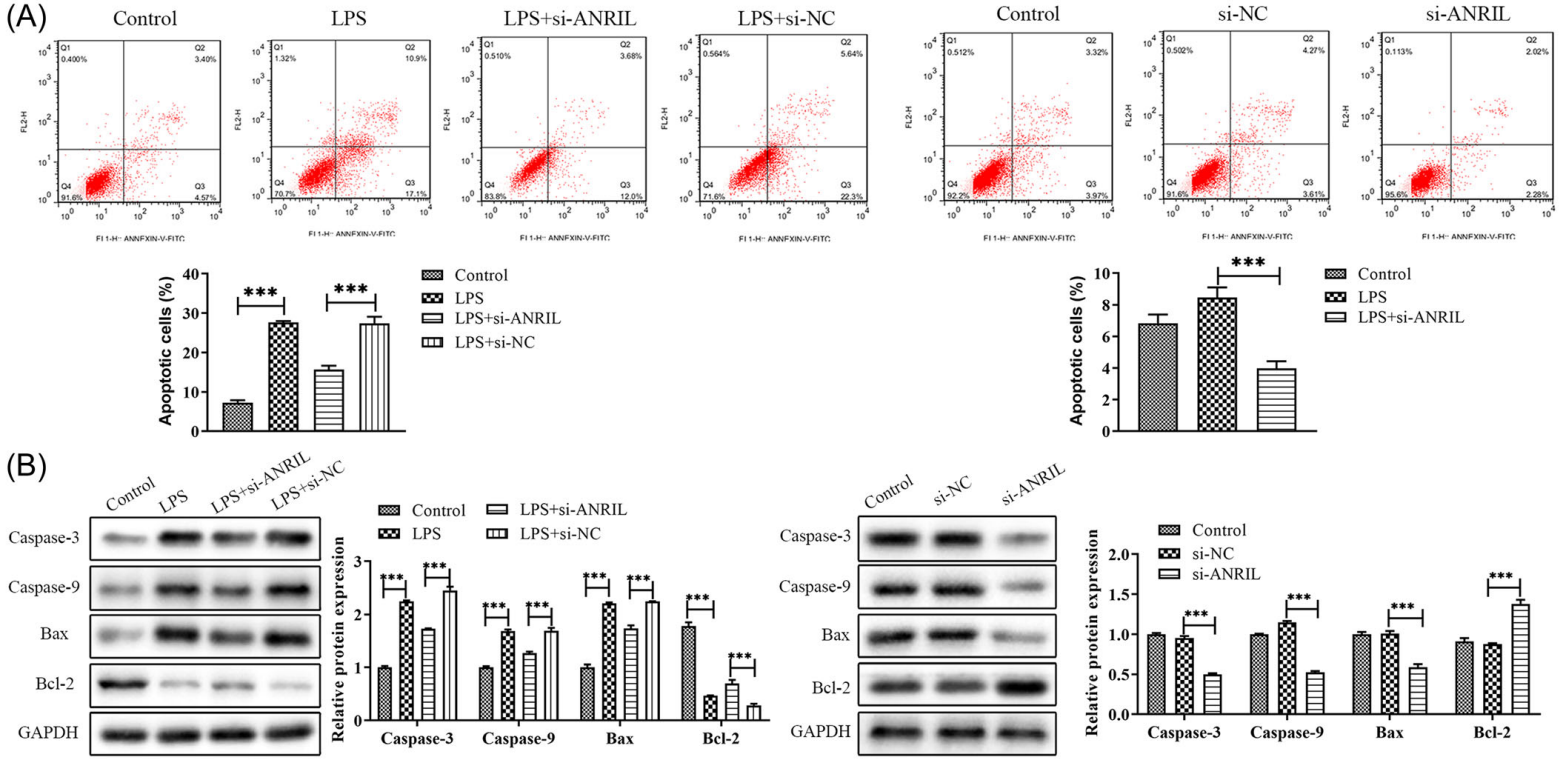
The effect of lnc‐antisense noncoding RNA in the INK4 locus (ANRIL) on cell apoptosis. (A) Flow cytometry was used to detect the activity of lnc‐ANRIL on cell apoptosis. (B) The expression levels of apoptosis‐related proteins caspase 3, caspase 9, Bcl‐2, and Bax were assessed by western blot. The experiments were replicated thrice and subjected to analysis using GraphPad Prism 5.0 software, employing analysis of variance in conjunction with Tukey's posthoc test. **p < .01, ***p < .001 compare with Control or lipopolysaccharide + si‐NC or si‐NC.

Flow cytometry was also conducted to investigate the lnc‐ANRIL role within MAC‐T cell apoptosis. The data in Figure 4A show that LPS caused more apoptosis compared with the control, while lnc‐ANRIL knockdown reduced the extent of apoptosis. Furthermore, the LPS + si‐ANRIL group showed a decrease in Caspase‐3, Caspase‐9, and Bax expressions and an increase in Bcl‐2 expression within MAC‐T cells compared with the LPS + si‐NC group (Figure 4B). Taken together, downregulation of lnc‐ANRIL expression reduced the MAC‐T cell apoptosis.

### 3.4 Downregulation of lnc‐ANRIL inhibits inflammatory responses and oxidative stress

**Figure 5:**
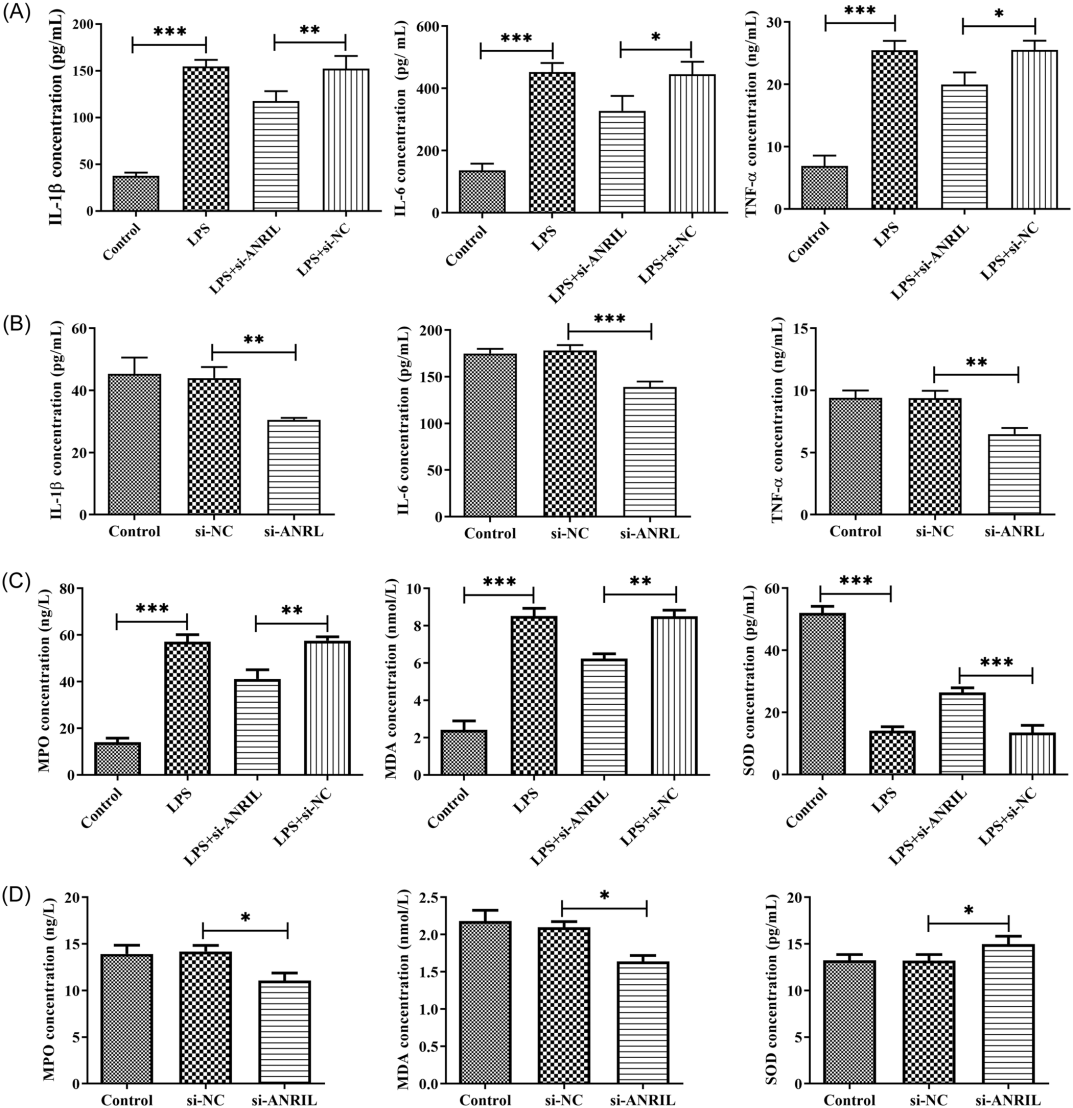
The effect of Lnc‐antisense noncoding RNA in the INK4 locus (ANRIL) on the inflammatory response and oxidative stress of mammary epithelial cell‐T cells. (A and B) ELISA assay was carried out to determine the effects of lnc‐ANRIL on the secretion of inflammatory factors. (C and D) Superoxide dismutase, myeloperoxidase, and malondialdehyde levels were detected using ELISA. The experiments were replicated thrice and subjected to analysis using GraphPad Prism 5.0 software, employing analysis of variance in conjunction with Tukey's posthoc test. *p < .05, **p < .01, ***p < .001 compare with Control or lipopolysaccharide + si‐NC or si‐NC.

ELISA was adopted for determining lnc‐ANRIL effects on the secretion of inflammatory factors (IL‐1β, IL‐6, and TNF‐α) and oxidative stress markers (SOD, MPO, and MDA). As shown in Figure 5A,B, LPS upregulated the expression of IL‐1β, IL‐6, and TNF‐α compared with the control group (*p* < .01). This means a more severe inflammation reaction. Besides, based on Figure 5A,B, lnc‐ANRIL downregulation alleviated IL‐1β, IL‐6, and TNF‐α production (*p* < .01). At the same time, LPS decreased SOD levels and increased MPO and MDA levels compared with the control group (*p* < .01), while inhibiting lnc‐ANRIL expression could reverse these changes (*p* < .01, Figure 5C,D). In general, downregulation of lnc‐ANRIL could inhibit the inflammatory response and oxidative stress in MAC‐T cells.

### 3.5 Silencing of lnc‐ANRIL expression inhibits the inflammatory response by regulating the NF‐κB axis

**Figure 6:**
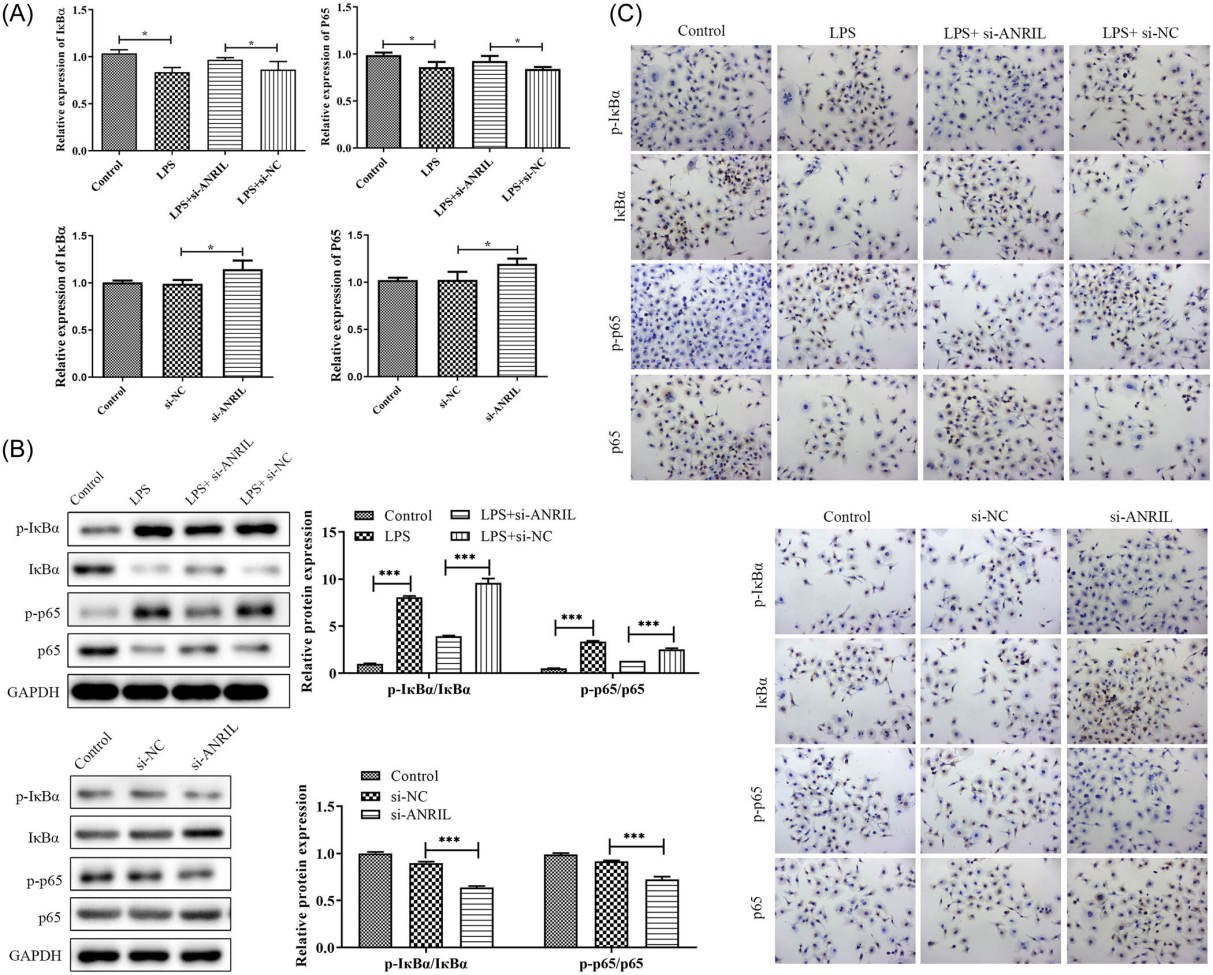
Silencing of lnc‐antisense noncoding RNA in the INK4 locus expression could inhibit inflammatory response by regulating the NF‐κB signaling pathway. (A) RT‐qPCR detection of mRNA expression levels of NF‐κB signaling pathway‐related genes (IκBα and p65). (B and C) Western blot and immunohistochemistry assays were performed to examine the expressions of the NF‐κB signaling pathway‐related proteins. The results were expressed as the mean ± SD of three independent experiments. The experiments were replicated thrice and subjected to analysis using GraphPad Prism 5.0 software, employing analysis of variance in conjunction with Tukey's posthoc test. **p < .01, ***p < .001 compare with Control or lipopolysaccharide + si‐NC or si‐NC.

NF‐κB is involved in inflammatory reactions. To further investigate the mechanism of lnc‐ANRIL in bovine MAC‐T, RT‐qPCR, western blot, and immunohistochemistry assays were conducted for examining NF‐κB signaling pathway‐related genes (IκBα and p65) levels. The results of Figure 6A indicate that knocking down lnc‐ANRIL significantly increases the expression level of IκBα and p65 mRNA. Based on Figure 6B,C, lnc‐ANRIL expression suppression significantly inhibited p‐IκBα and p‐p65 levels in LPS‐induced MAC‐T cells (*p* < .01). Nevertheless, p‐IκBα and p‐p65 levels were increased in the LPS group (*p* < .01), suggesting that lnc‐ANRIL may further aggravate the inflammatory response through regulating NF‐κB signaling pathway.

## 4 DISCUSSION

As an oncogene, lnc‐ANRIL has been extensively studied in many diseases like hepatocellular carcinoma, gastric cancer, prostate cancer, and diabetes mellitus.^20^, ^21^, ^22^, ^23^ However, the role and exact mechanisms of lnc‐ANRIL in bovine mastitis are still unclear. Understanding lnc‐ANRIL effects and underlying mechanisms in bovine MAC‐T under inflammatory conditions help determine a new target for the treatment of bovine mastitis. Here, we investigated the functional role and possible mechanisms of lnc‐ANRIL by experiments performed in MAC‐T cells.

Transient transfection of siRNA is a common strategy for studying gene functions.^24^ The Exosome Inc‐AFTR has the potential to exert anti‐inflammatory and antiapoptotic effects on mastitis cells MAC‐T through the inhibition of the TNF signaling pathway and the mitogen‐activated protein kinases signaling pathway.^25^ The interference of long noncoding RNA ANRIL was observed to decrease the occurrence of apoptosis in myocardial cells through the IL‐33/ST2 pathway. In vivo, experiments conducted on acute myocardial infarction mice revealed that the interference of long noncoding RNA ANRIL alleviated myocardial cell apoptosis and enhanced heart function.^26^ The knockout of the lncRNA ANRIL gene demonstrates a substantial enhancement in the proliferation and tube formation of human umbilical vein endothelial cells (HUVEC), while simultaneously suppressing inflammatory activation and apoptosis.^27^ However, the mechanism of lncRNA ANRIL in mastitis has not been reported. In our study, by exploring the function of lnc‐ANRIL, MAC‐T cells induced with LPS and transfected with siRNA‐ANRIL, and transfection efficiency could be determined under a fluorescence microscope. Further, we verified the effect of lnc‐ANRIL on cell proliferation. According to the results of CCK‐8, colony formation, and EdU assays, LPS could significantly suppress MAC‐T cell proliferation. After transfected with si‐ANRIL, MAC‐T cell proliferation was induced, compared with the LPS and LPS + si‐NC group. These data suggested the negative association between lnc‐ANRIL levels and cell proliferation. In addition, studies have shown that lncRNA ANRIL triggers myocardial cell apoptosis in acute myocardial infarction through IL‐33/ST2 signal transduction.^28^ Long‐chain noncoding RNA ANRIL regulates apoptosis in human trabecular meshwork cells by targeting microRNA‐7.^29^ Similarly, in our study, we also found that lnc‐ANRIL can induce apoptosis in MAC‐T cells.

Previous evidence demonstrated that inflammatory cytokines could act as mediators in cell functions.^30^, ^31^, ^32^, ^33^ The study revealed that ANRIL exerted an influence on the transcription levels of genes associated with the inflammatory response, NF‐κB signaling pathway, type I interferon‐mediated signal transduction pathway, and innate immune response in HUVEC.^34^ Consistent with the literature, this research found that LPS caused increases in IL‐1β, IL‐6, and TNF‐α expressions, and si‐ANRIL could alleviate this phenomenon, which meant the alleviation of inflammation. Moreover, the knockdown of lnc‐ANRIL released the oxidative stress induced by LPS. Lnc‐ANRIL exerts a crucial role in inflammatory response initiation. This induction occurs through the phosphorylation of Iκκ, further leading to NF‐κB activation, an important transcription factor regulating inflammatory cytokine expressions and secretion.^35^, ^36^, ^37^, ^38^ In this study, knockdown of lnc‐ANRIL expression significantly inhibited p‐IκBα and p‐p65 expressions in MAC‐T cells relative to the LPS group, indicating that lnc‐ANRIL might further aggravate the inflammatory responses through regulating the NF‐κB axis. The observation results show that the establishment by lnc‐ANRIL may through regulating mastitis of mammary gland epithelial cells of inflammation and oxidative stress level in the future play a role in clinical treatment of bovine mastitis.

However, there are still some limitations in this paper. First, in the selection of cells, other mastitis cell lines should be selected for validation experiments in the future. Second, experiments were not carried out in vivo in this paper, one limitation of cell culture models is that they lack the three‐dimensional structure and tissue microenvironment found in vivo. Cells in culture are often grown as monolayers, which may not accurately represent the physiological conditions and interactions that occur in a tissue or organ. Additionally, the absence of immune cells and other cell types that are present in vivo can limit the interpretation of the results. so it is very necessary to build an animal model of mastitis for future research.

## 5 CONCLUSION

To sum up, lnc‐ANRIL knockdown exerted its anti‐inflammatory response effects in LPS‐stimulated MAC‐T cells by modulation of the NF‐κB pathway. This suggests that lnc‐ANRIL may be a potential therapeutic target for mastitis treatment. This will provide valuable insights into the mechanism involved in bovine mastitis. Additionally, it presents a novel therapeutic approach for the clinical management of bovine mastitis in dairy establishments.

## AUTHOR CONTRIBUTIONS

Wei Lu, Jiang Lu, Jing Liu, and Jinye Lu performed the experiment and data analysis. Beibei Gu wrote the first draft of the article. Jinye Lu finalized the manuscript. All authors read and approved the final manuscript.

## CONFLICT OF INTEREST STATEMENT

The authors declare no conflict of interest.

## ETHICS STATEMENT

The authors agree to publication in the Journal.

## Data Availability

All data generated or analyzed during this study are available from the corresponding author upon reasonable request.

## References

[1] Fukata M , Vamadevan AS , Abreu MT . Toll‐like receptors (TLRs) and nod‐like receptors (NLRs) in inflammatory disorders. Sem Immunol. 2009;21(4):242‐253. 10.1016/j.smim.2009.06.005 19748439

[2] Kolattukudy PE , Niu J . Inflammation, endoplasmic reticulum stress, autophagy, and the monocyte chemoattractant protein‐1/CCR2 pathway. Circ Res. 2012;110(1):174‐189. 22223213 10.1161/CIRCRESAHA.111.243212 PMC3265021

[3] Porcherie A , Cunha P , Trotereau A , et al. Repertoire of *Escherichia coli* agonists sensed by innate immunity receptors of the bovine udder and mammary epithelial cells. Vet Res. 2012;43:14. 22330199 10.1186/1297-9716-43-14 PMC3305352

[4] Cha E , Kristensen AR , Hertl JA , et al. Optimal insemination and replacement decisions to minimize the cost of pathogen‐specific clinical mastitis in dairy cows. J Dairy Sci. 2014;97(4):2101‐2117. 24534495 10.3168/jds.2013-7067

[5] Nobrega DB , De Buck J , Naqvi SA , et al. Comparison of treatment records and inventory of empty drug containers to quantify antimicrobial usage in dairy herds. J Dairy Sci. 2017;100(12):9736‐9745. 28987586 10.3168/jds.2017-13116

[6] Herriges MJ , Swarr DT , Morley MP , et al. Long noncoding RNAs are spatially correlated with transcription factors and regulate lung development. Genes Dev. 2014;28(12):1363‐1379. 24939938 10.1101/gad.238782.114 PMC4066405

[7] Yang G , Lu X , Yuan L . LncRNA: a link between RNA and cancer. Biochim Biophys Acta. 2014;1839(11):1097‐1109. 25159663 10.1016/j.bbagrm.2014.08.012

[8] Grammatikakis I , Panda AC , Abdelmohsen K , Gorospe M . Long noncoding RNAs(lncRNAs) and the molecular hallmarks of aging. Aging. 2014;6(12):992‐1009. 25543668 10.18632/aging.100710 PMC4298369

[9] Sun CC , Li SJ , Li G , Hua RX , Zhou XH , Li DJ . Long intergenic noncoding RNA 00511 acts as an oncogene in non‐small‐cell lung cancer by binding to EZH2 and suppressing p57. Mol Ther Nucleic Acids. 2016;5(11):e385. 27845772 10.1038/mtna.2016.94 PMC5155326

[10] Li X , Wang H , Zhang Y , et al. Overexpression of lncRNA H19 changes basic characteristics and affects immune response of bovine mammary epithelial cells. PeerJ. 2019;7:e6715. 30984483 10.7717/peerj.6715 PMC6452850

[11] Wang H , Wang X , Li X , et al. A novel long non‐coding RNA regulates the immune response in MAC‐T cells and contributes to bovine mastitis. FEBS J. 2019;286(9):1780‐1795. 30771271 10.1111/febs.14783

[12] Saliminejad K , Khorram Khorshid HR , Soleymani Fard S , Ghaffari SH . An overview of microRNAs: biology, functions, therapeutics, and analysis methods. J Cell Physiol. 2019;234(5):5451‐5465. 30471116 10.1002/jcp.27486

[13] Li X , Huang TL , Zhang GD , Jiang JT , Guo PY . LncRNA ANRIL impacts the progress of osteoarthritis via regulating proliferation and apoptosis of osteoarthritis synoviocytes. Eur Rev Med Pharmacol Sci. 2019;23(22):9729‐9737. 31799639 10.26355/eurrev_201911_19535

[14] Ye S , Zhu S , Feng L . LncRNA ANRIL/miR‐125a axis exhibits potential as a biomarker for disease exacerbation, severity, and inflammation in bronchial asthma. J Clin Lab Anal. 2020;34(3):e23092. 31821602 10.1002/jcla.23092 PMC7083478

[15] Hu J , Wu H , Wang D , Yang Z , Dong J . LncRNA ANRIL promotes NLRP3 inflammasome activation in uric acid nephropathy through miR‐122‐5p/BRCC3 axis. Biochimie. 2019;157:102‐110. 30347231 10.1016/j.biochi.2018.10.011

[16] Zhou B , Li L , Qiu X , Wu J , Xu L , Shao W . Long non‐coding RNA ANRIL knockdown suppresses apoptosis and pro‐inflammatory cytokines while enhancing neurite outgrowth via binding microRNA‐125a in a cellular model of Alzheimer's disease. Mol Med Rep. 2020;22(2):1489‐1497. 32626959 10.3892/mmr.2020.11203 PMC7339647

[17] Gholami L , Ghafouri‐Fard S , Mirzajani S , et al. The lncRNA ANRIL is down‐regulated in peripheral blood of patients with periodontitis. Noncoding RNA Res. 2020;5(2):60‐66. 32346660 10.1016/j.ncrna.2020.04.001 PMC7182695

[18] Mirza AH , Berthelsen CH , Seemann SE , et al. Transcriptomic landscape of lncRNAs in inflammatory bowel disease. Genome Med. 2015;7(1):39. 25991924 10.1186/s13073-015-0162-2 PMC4437449

[19] Wang N , Zhu Y , Li D , et al. 2‐Methyl nonyl ketone from *Houttuynia Cordata* thunb alleviates LPS‐induced inflammatory response and oxidative stress in bovine mammary epithelial cells. Front Chem. 2022;9:793475. 35174140 10.3389/fchem.2021.793475 PMC8842123

[20] Deng W , Wang J , Zhang J , Cai J , Bai Z , Zhang Z . TET2 regulates LncRNA‐ANRIL expression and inhibits the growth of human gastric cancer cells. IUBMB Life. 2016;68(5):355‐364. 27027260 10.1002/iub.1490

[21] Zhang B , Wang D , Ji TF , Shi L , Yu JL . Overexpression of lncRNA ANRIL up‐regulates VEGF expression and promotes angiogenesis of diabetes mellitus combined with cerebral infarction by activating NF‐κB signaling pathway in a rat model. Oncotarget. 2017;8(10):17347‐17359. 28060742 10.18632/oncotarget.14468 PMC5370045

[22] Zhao B , Lu YL , Yang Y , et al. Overexpression of lncRNA ANRIL promoted the proliferation and migration of prostate cancer cells via regulating let‐7a/TGF‐β1/Smad signaling pathway. Cancer Biomark. 2018;21(3):613‐620. 29278879 10.3233/CBM-170683 PMC5859458

[23] Ma J , Li T , Han X , Yuan H . Knockdown of LncRNA ANRIL suppresses cell proliferation, metastasis, and invasion via regulating miR‐122‐5p expression in hepatocellular carcinoma. J Cancer Res Clin Oncol. 2018;144(2):205‐214. 29127494 10.1007/s00432-017-2543-y PMC11813335

[24] Wang XW , Hao J , Guo WT , et al. A DGCR8‐independent stable MicroRNA expression strategy reveals important functions of miR‐290 and miR‐183‐182 families in mouse embryonic stem cells. Stem Cell Reports. 2017;9(5):1618‐1629. 28988987 10.1016/j.stemcr.2017.08.027 PMC5830984

[25] Chen Y , Yang J , Huang Z , et al. Exosomal lnc‐AFTR as a novel translation regulator of FAS ameliorates *Staphylococcus aureus*‐induced mastitis. Biofactors. 2022;48(1):148‐163. 34855261 10.1002/biof.1806

[26] Yang J , Huang X , Hu F , Fu X , Jiang Z , Chen K . LncRNA ANRIL knockdown relieves myocardial cell apoptosis in acute myocardial infarction by regulating IL‐33/ST2. Cell Cycle. 2019;18(23):3393‐3403. 31674275 10.1080/15384101.2019.1678965 PMC6927712

[27] Liu X , Li S , Yang Y , et al. The lncRNA ANRIL regulates endothelial dysfunction by targeting the let‐7b/TGF‐βR1 signalling pathway. J Cell Physiol. 2021;236(3):2058‐2069. 32783191 10.1002/jcp.29993

[28] Chen H , Huang S , Guan F , et al. Targeting circulating lncRNA ENST00000538705.1 relieves acute coronary syndrome via modulating ALOX15. Dis Markers. 2022;2022:1‐24. 10.1155/2022/8208471 PMC9106501 35571613

[29] Zhao J , Sun H , Zhang JM , Wang M , Du XJ , Zhang JL . Long non‐coding RNA ANRIL down‐regulates microRNA‐7 to protect human trabecular meshwork cells in an experimental model for glaucoma. Eur Rev Med Pharmacol Sci. 2019;23(8):3173‐3182. 31081068 10.26355/eurrev_201904_17675

[30] Fathima Hurmath K , Ramaswamy P , Nandakumar DN . IL‐1β microenvironment promotes proliferation, migration, and invasion of human glioma cells. Cell Biol Int. 2014;38(12):1415‐1422. 25053165 10.1002/cbin.10353

[31] Bharti R , Dey G , Ojha PK , et al. Diacerein‐mediated inhibition of IL‐6/IL‐6R signaling induces apoptotic effects on breast cancer. Oncogene. 2016;35(30):3965‐3975. 26616855 10.1038/onc.2015.466

[32] Faunce DE , Gregory MS , Kovacs EJ . Effects of acute ethanol exposure on cellular immune responses in a murine model of thermal injury. J Leukoc Biol. 1997;62(6):733‐740. 9400814 10.1002/jlb.62.6.733

[33] Cuomo F , Coppola A , Botti C , et al. Pro‐inflammatory cytokines activate hypoxia‐inducible factor 3α via epigenetic changes in mesenchymal stromal/stem cells. Sci Rep. 2018;8(1):5842. 29643458 10.1038/s41598-018-24221-5 PMC5895792

[34] Wufuer A , Luohemanjiang X , Du L , et al. ANRIL overexpression globally induces expression and alternative splicing of genes involved in inflammation in HUVECs. Mol Med Rep. 2022;27(2):27. 36524379 10.3892/mmr.2022.12915 PMC9813546

[35] Fu Y , Zhou E , Liu Z , et al. *Staphylococcus aureus* and *Escherichia coli* elicit different innate immune responses from bovine mammary epithelial cells. Vet Immunol Immunopathol. 2013;155(4):245‐252. 24018311 10.1016/j.vetimm.2013.08.003

[36] Baker RG , Hayden MS , Ghosh S . NF‐κB, inflammation, and metabolic disease. Cell Metab. 2011;13(1):11‐22. 21195345 10.1016/j.cmet.2010.12.008 PMC3040418

[37] Hoesel B , Schmid JA . The complexity of NF‐κB signaling in inflammation and cancer. Mol Cancer. 2013;12:86. 23915189 10.1186/1476-4598-12-86 PMC3750319

[38] Allen IC , Moore CB , Schneider M , et al. NLRX1 protein attenuates inflammatory responses to infection by interfering with the RIG‐I‐MAVS and TRAF6‐NF‐κB signaling pathways. Immunity. 2011;34(6):854‐865. 21703540 10.1016/j.immuni.2011.03.026 PMC3166771

