# Correction to “Lnc‐ANRIL Modulates the Immune Response Associated With NF‐κB Pathway in LPS‐Stimulated Bovine Mammary Epithelial Cells”

**DOI:** 10.1002/iid3.70308

**Published:** 2025-12-18

J. Lu, B. Gu, W. Lu, J. Liu, and J. Lu, “Lnc‐ANRIL Modulates the Immune Response Associated With NF‐κB Pathway in LPS‐Stimulated Bovine Mammary Epithelial Cells.” *Immunity, Inflammation and Disease* 11, no. 12 (2023): e1125, https://doi.org/10.1002/iid3.1125.

In Figure 4B of this paper, the Caspase‐3 bands were mistakenly duplicated from the P27 bands shown in Figure 3B. The authors have provided the original image for the Caspase‐3 bands and corrected the figure. They confirm that all experimental results and corresponding conclusions in the paper remain unaffected. The corrected Caspase‐3 bands and the updated Figure 4 are shown below.

Corrected caspase ‐ 3 bands
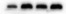


Corrected Figure 4

**Figure:**
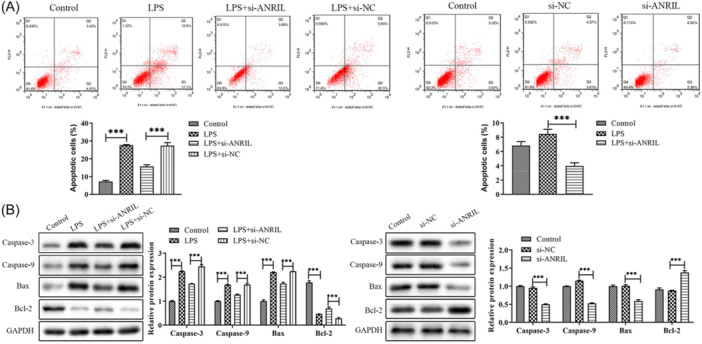
FIGURE 4 |

The authors apologize for this error.

